# Predicting the time to get back to work using statistical models and machine learning approaches

**DOI:** 10.1186/s12874-024-02390-4

**Published:** 2024-11-29

**Authors:** George Bouliotis, M. Underwood, R. Froud

**Affiliations:** 1 Warwick Clinical Trials Unit, University of Warwick, Coventry, UK; 2 Høyskolen Kristiania, Oslo, Norway

**Keywords:** Machine Learning, Survival analysis, Statistical methods, Return to work, Socioeconomic deprivation

## Abstract

**Background:**

Whether machine learning approaches are superior to classical statistical models for survival analyses, especially in the case of lack of proportionality, is unknown.

**Objectives:**

To compare model performance and predictive accuracy of classic regressions and machine learning approaches using data from the Inspiring Families programme.

**Methods:**

The Inspiring Families programme aims to support members of families with complex issues to return to work. We explored predictors of time to return to work with proportional hazards (Semi-Parametric Cox in Stata) and (Flexible Parametric Parmar-Royston in Stata) against the Survival penalised regression with Elastic Net penalty (scikit-survival), (conditional) Survival Forest algorithm (pySurvival), and (kernel) Survival Support Vector Machine (pySurvival).

**Results:**

At baseline we obtained data on 61 binary variables from all 3161 participants. No model appeared superior, with a low predictive power (concordance index between 0.51 and 0.61). The median time for finding the first job was about 254 days. The top five contributing variables were ‘family issues and additional barriers’, ‘restriction of hours’, ‘available CV’, ‘self-employment considered’ and ‘education’. The Harrell’s Concordance index was range from 0.60 (Cox model) to 0.71 (Random Survival Forest) suggesting a better fit for the machine learning approaches. However, the comparison for predicting median time on a selected scenario based showed only minor differences.

**Conclusion:**

Implementing a series of survival models with and without proportional hazards background provides a useful insight as well as better interpretation of the coefficients affected by non-linearities. However, that better fit does not translate to substantially higher predictive power and accuracy from using machine learning approaches. Further tuning of the machine learning algorithms may provide improved results.

**Supplementary Information:**

The online version contains supplementary material available at 10.1186/s12874-024-02390-4.

## Introduction

Good work is positively associated with good health [1]. For many, a job is an essential part of self-identity. Improving work participation can improve health outcomes, quality of life, well-being, and reduce poverty [2]. It is not clear how best to help some unemployed groups. Whilst interventions such as Individual Placement and Support (IPS) – a highly structured supported employment intervention that follows a ‘place-then-train’ model – is more than twice as likely to lead to employment, compared to traditional vocational rehabilitation, for people with severe mental health difficulties, there is little evidence on how best to help people who have a history of crime or anti-social behaviour in their families [3]. People who are unemployed and who are also from backgrounds in which there is history of crime or anti-social behaviour within families, may face additional, or different, obstacles to gaining or returning to work. Supported employment programmes generally may be of some benefit; these have also been shown to be effective in people with a range of different health and social problems [4]. It is not known if there are any particular characteristics that predict employment outcomes in people with complex family issues, who are part of a supported employment programme.

Logistic or Cox regression approaches have been widely used for modelling outcome in such studies, where outcomes are typically categorical (e.g. whether a person achieves employment by a certain definition) or time-to-event (e.g. how long it takes a person to achieve employment by a certain definition) [5]. A rise in popularity of artificial intelligence, or machine learning techniques has resulted in increasingly diverse applications of these techniques Comparisons of predictive model performances may be useful to researchers in applied health and social care. In contrast to regression, machine learning algorithms make fewer assumptions about data structure [6]. Several comparisons exist between machine learning algorithms and traditional logistic/multinomial logit regression, demonstrating approaches can yield similar performance, and highlighting a risk of overfitting in machine learning techniques [7]. A 2021 comparison between machine learning algorithms and linear regression for continuous outcomes in health data sets, found regression is less prone to overfitting and out-performs commonly used machine learning techniques unless truly non-linear or heteroscedastic relationships are present [8]. Some comparisons between Cox regression and machine learning techniques have been made, but further, and population-specific research is needed [7].

Our aim was to use both conventional statistical and machine learning techniques to identify predictors of return to work in those with complex family issues. We were interested in both: assessing the model performance including accuracy and prediction, including comparing, and contrasting the findings from those models and identifying factors promoting or impeding return to work in customers of a supported employment programme.

## Methods

### Participants

The Inspiring Families programme (https://www.serco-ese.com/inspiring-families) was a partnership between the Department of Work and Pensions and Serco International PLC. It aimed to help people from families that may have complex family issues, including for example where there is a history of crime, substance dependence, or anti-social behaviour. It was open to people legally resident in the UK, with a right to work, aged 16 or over, who self-declared criteria that led to difficulty in gaining employment. Participants lived mainly in north and east London. The intervention began in January 2017 and we had access to data up until a point of freezing the dataset for transfer to University of Warwick in June, 2019.

People were referred by the staff from the Department of Work and Pensions (DWP) to Serco. If people wished to join the programme, they were invited to attend a one-hour (minimum) face-to-face Initial Engagement meeting within five days of a referral from DWP. At this meeting, an initial needs assessment was initiated by a personal adviser, who also conducted a financial ‘better-off in-work’ calculation for the participant. At this time, advisors collected basic demographic data and customers completed a 32-item questionnaire focused on family circumstances, with many binary variables, such as whether there was a history of drug/alcohol abuse, convictions, the ability to drive, etc., and obstacles to getting a job identifying potential challenges customers might face when looking for a job (Table 1 & Appendix, Table S1). It was identifying which of these factors are most important for predicting return to work that was the focus of this project. The customers’ consent to share information with others where appropriate, and consent for Serco to contact employers during the programme as participants find work, was sought during this meeting.

The needs assessment included a series of detailed questions about the participants’ work-history and discussions around an Initial Action Plan commenced based on the identified needs. The Action Plan commenced at this first meeting but could be completed at one of the subsequent follow-up meetings (below). The Action Plan set out details of what it was assessed needed to be done, who needed to do it and by when. It documented plans for support with family issues, facilitation of access to vacancies, job search support, personal coaching to build motivation and confidence, help with CV preparation, help with economic calculations, and advice on other supporting services. Personal advisers aimed to contact participants once per fortnight as a minimum with increased contact as required on a case-by-case basis. Action Plans were regularly reviewed, and actions were recorded as completed as soon as these were identified. Our analyses focus on the first date of work starting from the first interview, i.e., the start of the Initial Action Plan.

### Analysis

Our analysis plan was to compare and contrast the results from two statistical time-to-event regressions and two machine learning approaches. Since machine learning algorithms lose efficiency with missing values, and the multiple imputation methods are yet to be integrated, we analysed only an imputed dataset using the Hot Deck method [9, 10]. Nevertheless, we recognise the superiority of the multiple imputation approach for dealing effectively with missing values and the limitations of the Hot-Deck method in complying with the Rubin’s approach [11]. The rationale behind our combinatory approach (statistical and machine learning algorithms methods) was to find out if using machine learning approaches would lead to additional insights into which prognostic variables affect the employability of programme participants, when compared to well-established statistical models. We evaluated the performance of three conventional analytical models and two machine learning approaches on this dataset. Our outcome of interest was the date of starting their first job after joining programme.

Initially, and before implementing any model, we derived Kaplan Meier curves and assessed linearity using Martingale residuals. Then, we checked proportionality using visual assessments and assessed linearity. Then, we checked proportionality using visual assessments and the Schoenfeld residuals test for proportional hazards.

#### Semi-Parametric Regression - Cox

This model has been used for analysing time-to-event data since the 1970s [12, 13]. Although the semi-parametric approach adds flexibility of this model by allowing the baseline hazard function to be unspecified, there are two strict model assumptions: The hazard **ratio** is assumed to be constant over time and the survivorship effect is proportional over time. Deviation from this proportionality assumption for the hazard increases the chance of bias, thus accommodation of time-varying effect(s) is suboptimal. The model is not robust to the proportional-hazards assumption deviation and in this case, stratification should be used [14].

#### Flexible-Parametric (Royston-Parmar) Regression

The distributional specification of the baseline hazard is a more theory-driven alternative as the (typical) step function approximation of the event time improves model fit. This model supports a viable alternative of a flexible specification using splines for a smoothed parameterisation of the baseline hazard [15, 16].

The major features of this model are prediction flexibility due to adjustment for covariates, especially the time-varying covariates, more transparent and more efficient handling of the time-varying covariates. It is suggested that the standard (non-tuned) model specification should be the hazard-scaled model with a three degrees of freedom spline for the spline function that represents the baseline (log cumulative-hazard) function [16]. This is the two-knots restricted cubic spline function. For the time-dependent covariates one degree of freedom is used.

#### Cox elastic net

Use of penalised regression has been advocated for a long time as it provides a good support for model parsimony. Where a large number of variables are analysed in a model, a method to remove non-contributing variables is very useful. Briefly, the elastic net approach combines two individual regression penalties known as lasso (l_1_) and ridge regression (l_2_) borrowing strength from the flexibility of both approaches. The balance of those two penalties is expected to result to a better model performance. Here, we use this regularised regression to ease selection of the important variables in predicting job uptake [17, 18].

#### Random survival forest

These (semi) parametric approaches are known to perform adequately in time to event studies of average size and few variables where the interest is primarily on pre-specified variables as either factors or interactions, commonly adjusted for selected co-variate [19]. However, when the number of covariates increases substantially, likelihood-based optimisation algorithms underperform, sometimes substantially [20]. On the other hand, classic frequentist inference may be of secondary importance compared to predicting who is going to experience the event. Constrained by numerous *p*-value based inefficiencies, data analysts are looking for big-data alternatives that can handle numerous covariates simultaneously. Machine learning approaches are expected to perform more efficiently within this high-dimensional data context, either by employing proportional hazard or less restrictive alternatives. Here, we use the Random Survival Forest as an ensemble algorithm that is based on bagging of classification trees for survival data [21, 22]. We expected that the advantages of this fully data-driven approach would have positive effects on variable selection and event occurrence, avoiding common regression issues such convergence, heteroskedasticity and covariates’ correlational influences.

#### Survival support vector machine

This is a flexible machine learning algorithm that does not rely on asymptotic properties, although it involves statistical learning theory. As a classifier, support vector machine ‘clusters’ observations so to provide the ‘high margin‘, that is the threshold between the clusters that can accurately classify observations into groups/classes of interest [23]. In other words, the aim of this algorithm is to find a classification boundary that maximises the distance from the nearest observations of all classes. It is this optimal boundary, known as maximum margin hyperplane, that makes the algorithm a useful analysis tool. The larger the margin, the lower the error as the boundary is less sensitive to outliers and noise. However, like other models, support vector machine is frequently subject to overfitting.

#### Implementation software

Two programming languages were used to implement the analyses reported here. The data preparation for analysis and the statistical modeling was implemented in Stata ( Stata v.17, StataCorp. 2022, College Station, TX: Stata Press). The machine learning models were fitted using python v2.8 and the libraries ( pysurvival 0.1.2 2019, scikit-survival 0.19.0 2022, matplotlib 3.6 2022) [24, 26]. We validated the multi-level models using the ‘train_test_split’ function from the library scikit-learn (1.1.3), splitting the dataset 75/25:training/test dataset [27]. We did not validate the semi-parametric and flexible parametric models because the unreliability of validation for time to event models with high levels of censoring [28, 29].

## Results

We obtained data from 3161 people. Participants’ characteristics and questionnaire data collected at the time of the first meeting with an advisor to develop an Action Plan are in Appendix 1 Table S1. We had follow-up data for a median of 715 days with a maximum of 1,314 days. Around 29% (906/3161) of participants got a first job within this time.

Multivariable survival models revealed the following statistically significant factors associated with the job finding: previous employment, fewer restriction on working time, absence of physical/mental health problems, not using non-prescribed drugs, having a criminal record, reported domestic violence, ability to use a computer, having a written CV available, not wanting to be self-employed, no caring responsibilities, and needing practical financial help (Tables S2 and S3).

**Table 1:** Participant characteristics and how these predict getting a job

Variables	*n* (Got a job)	Breakdown by category	*P*-value χ2
n (%)	906 (29)	3161 (100)	
Age Group, n (%)			
16 to 25	214 (31)	684 (22)	
26 to 35	240 (29)	820 (26)	
36 to 45	253 (31)	821 (26)	
46 to 55	131 (24)	537 (17)	
56 to 69	68 (23)	299 (9)	0.01
Ethnicity, n (%)			
White	281 (27)	1023 (32)	
Black	319 (29)	1093 (35)	
Asian	167 (27)	609 (19)	
unknown/other	139 (32)	436 (14)	0.32
Highest Educational Attainment, n (%)^b^			
Below primary ISCED 0^a^	10 (18)	57 (2)	
primary ISCED 1	22 (32)	68 (3)	
Lower Secondary ISCED 2	170 (28)	600 (23)	
Upper Secondary ISCED 3	321 (31)	1048 (39)	
Post Secondary ISCED 4	158 (27)	576 (22)	
Tertiary ISCED 5–8	112 (36)	310 (12)	0.03
Gender, n (%)			
Male	392 (29)	1364 (43)	
Female	514 (29)	1797 (57)	0.93
Living situation, n (%)			
Lives alone	143 (26)	547 (17)	
single parent	47 (29)	164 (5)	
with someone	376 (28)	1345 (43)	
children/dependent	207 (32)	657 (21)	
other/unknown	133 (30)	448 (14)	0.3
London Borough, n (%)			
east	730 (30)	2461 (78)	
north	139 (27)	515 (16)	
outer^c^	37 (20)	185 (6)	0.01
Household issues and barriers, n (%)			
additional barriers	427 (34)	1260 (40)	
long term unemployed	233 (33)	700 (22)	
involved in crime	133 (19)	696 (22)	
Unknown or other barrier	113 (22)	505 (16)	0.01

^a^ ISCED = International Standard Classification of Education

^b^ Missing data = 502 (16%)

^c^ Includes Greenwich

**Fig. 1:**
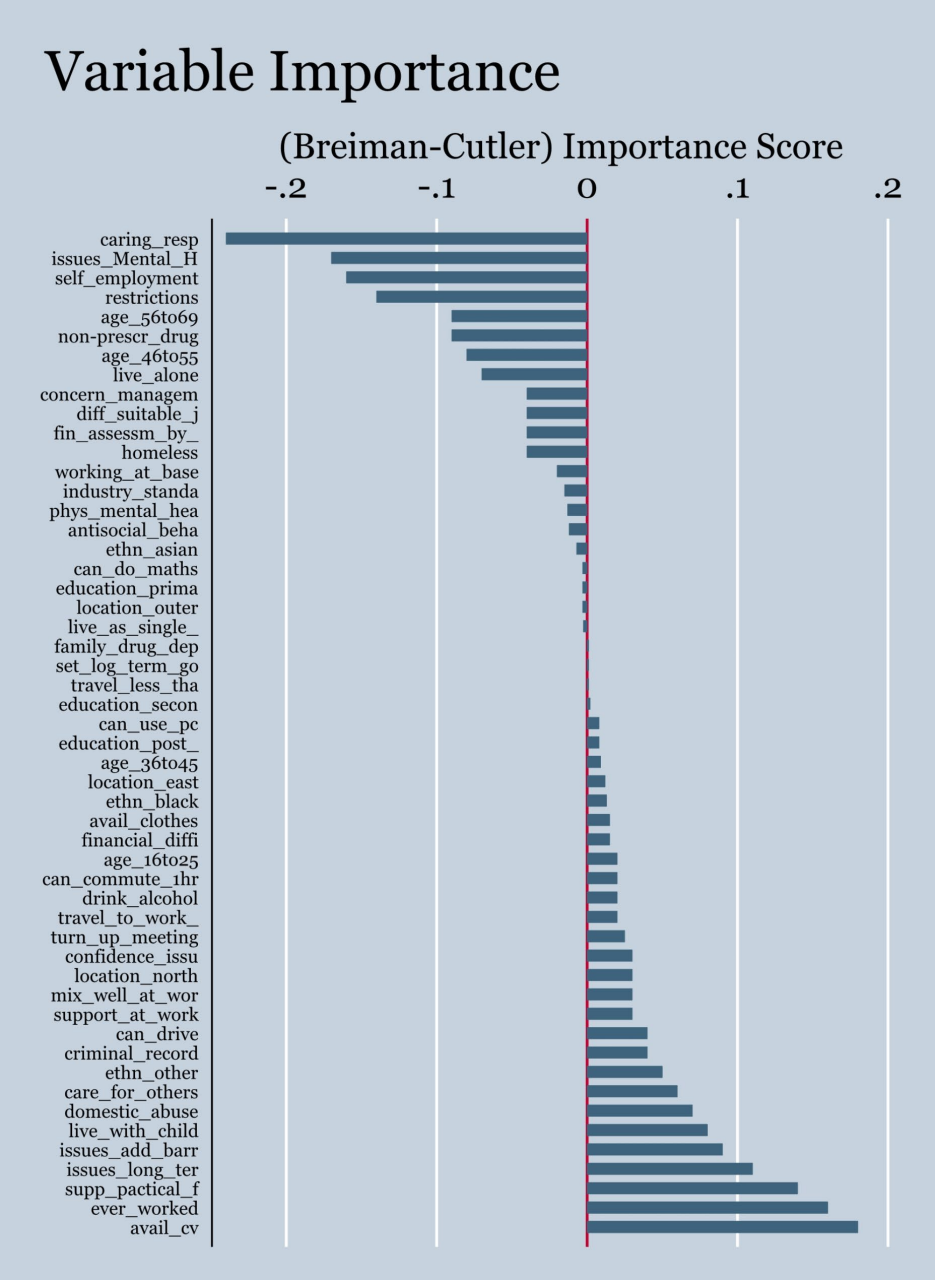
Variable Importance from the

The two proportional hazard models provided very similar results, as expected. They found a strong effect of 15 variables on getting a first job (Fig. 1, Appendix Tables S1-4). Among others, the five significant accelerating variables were ‘having issues in the household’, ‘had worked before’, experiencing ‘domestic abuse and violence’, being in the age-group 36–45 and having a written CV available. On the other hand, the variables that delayed job-getting were ‘using drugs’, ‘having caring responsibilities’, ‘considering self-employment’ and ‘having a restriction of hours’. Both models failed the proportionality assumption with four variables significantly deviating from proportionality. The Harrel’s concordance (C-index) was 0.64.

Instead of modifying the proportional hazard models to accommodate time-varying covariates and linearity issues, we focused on two machine learning approaches: To bridge the gap between the proportional hazard and the machine learning models, we implemented a penalty function to the Cox model by using the Elastic Net regression, by deriving a data-driven alpha weight (α). That alpha is the hyperparameter for shrinkage (towards zero). This indicates that ‘domestic abuse and violence’, ‘self-employment considered’, ‘caring responsibilities in the household’, use of drugs’ and ‘tertiary education’ are the most influential variables in predicting getting a first job (Fig. 1).

We then fitted two well-established machine learning algorithms that have been extended to accommodate censored data for time to event analyses.

The Survival Random Forest achieved a moderate performance. The C-index of the conditional (0.6) and the random (0.58) were comparable and the top five most contributory variables were ‘restriction of working hours’, ‘caring responsibilities of the customer’, ‘written CV available’, ‘health issues in the household’, and‘ past work’. Given the fact that the C-index for the Cox PH was 0.64, none of the survival random models was able to perform beyond that point.

The support vector machine algorithm, also achieved moderate performance. It suggested that the top five most important variables were ‘caring responsibilities‘, ’restriction of working hours‘, ‘available written CV‘, ‘working experience’, and ‘health issues‘ (Fig. 1). Although the Integrated Brier Score (IBS) was kept very low 0.02, far below the concerning 0.25 limit and the prediction error between observed and fitted values was low too (MAE = 0.284), the model’s performance was not promising, providing a low Harrel’s C-index score of 0.57. After, kernel extension tuning between the ’inverse multi-quadratic’ and ‘Gaussian‘, the achieved C-index increased to 0.60 and 0.90, respectively, however, at the cost of achieving partial convergence only. Even when we tried different kernel types, convergence warnings emerged indicating poor optimisation.

Finally, we derived the ‘feature’s importance’ graph for displaying the contribution of the variables in the model’s explanatory power (Fig. 1).

## Discussion

In this study, we analysed data from the ‘Inspiring Families’ programme for tackling unemployment among underprivileged households. Our analyses are focused on the first job taken since the customer’s enrolment. We found that several factors contribute to earlier job acquisition. Surprisingly, gender, ethnicity, location, and cohabitation (living with) did not predict earlier employment. As expected, both younger age and education accelerated job finding in a consistent manner, even though the latter did not reach statistical significance.

Also, key modifiable characteristics of the customer (i.e., availability of a written CV, practical financial skills, demonstrable working experience) found to be consistently important features of getting a (first) job earlier. Unsurprisingly, for those participants who were ‘labour market ready‘a favourable outcome is observed significantly earlier suggesting that improvements in that direction should be a priority.

Unmodifiable, or less modifiable, but nevertheless key characteristics such as ‘caring responsibilities at home’, ‘restrictive availability’, ‘drug abuse’, and ‘need for financial help’ predicted a delay to finding, and often a failure to find a job, during the follow up period. That these difficult to modify factors are preventing people from entering the labour market may explain why some potentially modifiable factors like the ability to use a computer and driving skills, the ability to read and write and do maths, etc… were not statistically significant predictors of return to work. Other factors, highly collinear with variables of similar nature, such as criminal convictions and a history of family drug or abuse issues were also not statistically significant.

Interestingly there was an association between the personal issues that made eligibility for the programme possible, and job uptake. Compared to crime-gang involvement, other types of issues like long term unemployment and additional barriers (to daily tasks) do improve first job uptake. Thus, demonstrating the importance of criminal convictions as an obstacle to securing employment. It was striking that all models consistently found out that domestic abuse within the household is associated with significantly earlier job finding, suggesting that the programme is responsive and supportive in ‘emergency’ situations, when most needed.

All of our models’ performances were moderate to poor. There are several reasons why this might be the case. Getting a (first) job is a multifactorial process when coming from an underprivileged household with rather complex set of interacting relationships. As the aim was to analyse the available interview information only, it may be hypothesised that such information is not powerful enough to predict job taking with a high accuracy. In other words, to attribute the observed job uptake delay to key factors, we need first to explicate the relationship and influence of the personal/family barriers and the job-finding features. On the other hand, the analysis provides supportive evidence about the impact of the programme to a substantial number of underprivileged London families. That nearly a third of participants were helped into work is an important observation.

Personal and family obstacles have a detrimental effect on coming back to the labour market after long-term unemployment that is frequently coming with antisocial behaviour. Some of these are potentially modifiable and important targets for future interventions.

### Limitations and further plans

This is an observational study based on survey data and as such it is prone to bias and related issues. Data quality, including missingness, is among those issues. In this case however, the large size of the sample and the systematic collection of both the outcome data and survey data increase the validity of the sample and the findings reported here.

Analysis issues due to models (mal/miss) fit did not affect the results as the contribution and the direction of the variables found to be highly consistent across the different models. It should be noted here, regarding the machine learning approaches, that some further tuning of the hyperparameters may be necessary for achieving reliable convergence under challenging situations (e.g. high multicollinearity). However, we provided here the results from only the models that achieved convergence.

Robust approaches to internal validation are not available for all of the models we tested. Nevertheless, this does not affect the need to carefully consider external validation before choosing to use machine learning models.

As a future plan, it would be worthwhile to generate a parsimonious model accompanied by a nomogram that could predict and score both time and type of employment that tailored to a participant needs. In terms of service delivery there may be merit in considering the difference between reputational barriers (e.g. past criminal convictions) and non-reputational obstacles (e.g. travel distance, caring activities) what differential approaches might help these groups secure work. We have also identified some key modifiable factors which, if addressed, might help people secure work. For example, short term training on numeracy, computing and job marketing skills.

## Conclusions

We did not find machine learning algorithms to be demonstrably superior to conventional regression models. Great care is needed in choice of analytical technique for studies of this nature.

## Electronic supplementary material

Below is the link to the electronic supplementary material.

Supplementary Material 1

## Data Availability

The confidential nature of the data used means it cannot be made freely available. Data sharing requests should be made to Serco Ltd.
