# Supplementary material for: Predicting the time to get back to work using statistical models and machine learning approaches
Source: BMC Med Res Methodol. 2024 Nov 29;24:295. doi: 10.1186/s12874-024-02390-4 (PMC11606207; doi:10.1186/s12874-024-02390-4)
Supplement: Supplementary file 1 — Supplementary Material 1 [file 12874_2024_2390_MOESM1_ESM.docx]

**Predicting the Time to get Back to Work using Statistical Models and Machine Learning Approaches**

**Table S1:** Participant questionnaire responses and how these predict getting a job

| Variables | Got a job  n (%) | Breakdown by category n (%) | P-value χ2 |
| --- | --- | --- | --- |
| Missing n(%) | 906 (29) | 3161 (100.0) |  |
|  |  |  |  |
| Employment Survey Questions | | | |
| Q.1 Is the customer homeless?, n (%) Missing 501 (16) | | | |
| No | 759 (30) | 2515 (95) |  |
| Yes | 2 (50) | 4 (<1) |  |
| Not disclosed | 32 (23) | 141 (5) | 0.11 |
| Q.2 Has the customer ever worked, n (%)  Missing 4 (<1) | | | |
| No | 177 (24) | 753 (24) |  |
| Yes | 727 (30) | 2404 (76) | <0.01 |
| Q.3 Is the customer working at present either in part-time, voluntary or permitted work?  Missing 5(<1) | | | |
| No | 805 (29) | 2790 (88) |  |
| Yes | 99 (27) | 366 (12) | 0.47 |
| Q.4 Do they have a long term job goal?), n (%) Missing = 6 (<1) | | | |
| No | 451 (28) | 1586 (50) |  |
| Yes | 453 (28) | 1569 (50) | 0.79 |
| Q.5 Does customer have any restrictions on the number of hours/days they can work each week? Missing 4 (<1) | | | |
| No | 589 (32) | 1864 (59) |  |
| Yes | 315 (24) | 1293 (41) | <0.01 |
| Q.6 Does the customer drive?, n (%)  Missing n 7 (<1) | | | |
| No | 722 (28) | 2546 (81) |  |
| Yes | 180 (30) | 608 (19) | 0.54 |
| Q.7: Does the customer have any physical or mental health problems? | | | |
| No | 490 (30) | 1612 (51.1) |  |
| Yes | 414 (27 | 1545 (48.9) | 0.03 |
| Q.8: Does the customer take non-prescribed drugs?, n (%)  Missing 88 (3) | | | |
| No | 856 (29) | 2977 (97) |  |
| Yes | 18 (19) | 96 (3) | 0.03 |
| Q.9: Does the customer drink alcohol?  Missing 100 (3) | | | |
| No | 667 (27) | 2426 (79) |  |
| Yes | 199 (31) | 635 (21) | 0.06 |
| Q.10: Does the customer have a criminal record?  Missing 5 (<1) | | | |
| No | 757 (28) | 2714 (86) |  |
| Yes | 146 (33) | 442 (14) | 0.03 |
| Q.11: Does the customer have their own (stable) long term accommodation (owned or rented?)  Missing 6 (<1) | | | |
| No | 162 (29) | 560 (18) |  |
| Yes | 741 (29) | 2595 (82) | 0.86 |
| Q.12: Does the customer have any caring responsibilities and/or provide ongoing support to another family member  Missing 4 (<1) | | | |
| No | 488 (29) | 1677 (53) |  |
| Yes | 416 (28) | 1480 (47) | 0.54 |
| Q.13: Do any family members have a drug or alcohol dependency? n (%)  Missing 94 (3%) | | | |
| No | 860 (28) | 3019 (98) |  |
| Yes | 13 (27) | 48 (2) | 0.83 |
| Q.13: Are there any antisocial behaviour issues within the household or family at present?  Missing 94 (3) | | | |
| No | 839 (28) | 2957 (96) |  |
| Yes | 33 (30) | 110 (4) | 0.71 |
| Q.14: Are there any domestic violence abuse issues within the household or family at present? Missing 85 (3) | | | |
| No | 832 (28) | 2964 (96) |  |
| Yes | 45 (40) | 112 (4) | 0.01 |
| Q.15: Does the customer or any family member have any financial difficulties, including ability to manage money or debt that you are struggling to pay off?  Missing 84 (3) | | | |
| No | 712 (28) | 2518 (81.8) |  |
| Yes | 164 (29) | 559 (18.2) | 0.61 |
| Q.16: Does the customer have any concerns other than those already disclosed either personally or within their family?  Missing 5 (<1) | | | |
| No | 783 (86.6) | 2731 (87) |  |
| Yes | 121 (13.4) | 425 (13) | 0.93 |
| Q.17: Is the customer comfortable with reading writing and everyday maths?  Missing 6 (<1) | | | |
| No | 81 (25) | 321 (10) |  |
| Yes | 823 (91.0) | 2834 (90) | 0.15 |
| Q.18: Is the customer able to use a computer independently to find and apply for jobs?  Missing 5 (<1) | | | |
| No | 135 (24) | 552 (17) |  |
| Yes | 769 (30) | 2604 (83) | 0.02 |
| Q.19: Is there a basic industry standard requirement needed for the job the customer is looking for (e.g. a CSCS card^a^?  Missing 8(<1) | | | |
| No | 634 (29) | 2171 (69) |  |
| Yes | 269 (27) | 982 (31) | 0.3 |
| Q.20: Does the customer have any confidence issues or difficulties with being able to motivate themselves (either in their day to day life or in terms of the job searching process?  Missing 5 (<1) | | | |
| No | 589 (29) | 2023 (64.1) |  |
| Yes | 314 (28) | 1133 (35.9) | 0.4 |
| Q.21: Does the customer have any difficulties finding suitable jobs to apply for using a variety of methods or applying for jobs (e.g. writing cover letters) ?  Missing 5(<1) | | | |
| No | 568 (30) | 1907 (60) |  |
| Yes | 336 (27) | 1249 (40) | 0.08 |
| Q.22: Does the customer have a CV  Missing 4 (<1) | | | |
| No | 239 (23) | 1057 (33) |  |
| Yes | 665 (32) | 2100 (67) | <0.01 |
| Q.23: Does the customer have clothing that would be suitable to wear for a job interview?  Missing 75 (2) | | | |
| No | 190 (28) | 683 (22) |  |
| Yes | 695 (29) | 2403 (78) | 0.57 |
| Q.24: Has the customer considered or are they looking for self-employment?  Missing 8 (<1) | | | |
| No | 826 (29) | 2813 (89) |  |
| Yes | 78 (23) | 340 (11) | 0.01 |
| Q.25: Would the customer like us to complete a financial assessment in order to show them how much better off in work they?  Missing 14 (<1) | | | |
| No | 387 (29) | 1344 (43) |  |
| Yes | 514 (29) | 1803 (57) | 0.86 |
| Q.26: Does the customer have any difficulties with or concerns over how they will manage their money when they start work?  Missing 96 (3) | | | |
| No | 825 (29) | 2889 (94) |  |
| Yes | 44 (25) | 176 (6) | 0.31 |
| Q.27: Is the customer able to turn up to appointments, meetings or activities (including employment) on time?  Missing 4 (,1) | | | |
| No | 16 (21) | 77 (2) |  |
| Yes | 888 (29) | 3080 (98) | 0.12 |
| Q.28: Does the customer have any concerns about their ability to mix with and get on well with work colleagues?  Missing 93 (3) | | | |
| No | 826 (29) | 2877 (94) |  |
| Yes | 43 (23) | 191 (6) | 0.07 |
| Q.29: Does the customer have any concerns about being able to get to and from work?  Missing 82 (3) | | | |
| No | 742 (28) | 2624 (85) |  |
| Yes | 129 (28) | 455 (15) | 0.97 |
| Q.30: Does the customer have any caring responsibilities where alternative arrangements will need to made when they are at work?  Missing 90 (3) | | | |
| No | 663 (30) | 2180 (71) |  |
| Yes | 207 (23) | 891 (29) | <0.01 |
| Q.31: Is there anything in particular that the customer may need support with whilst at work?  Missing 115 (4) | | | |
| No | 699 (28) | 2460 (81) |  |
| Yes | 168 (29) | 586 (19) | 0.90 |
| Q.32: Is there anything practical financial that the customer may need to be able to either start or stay in work?  Missing 116 (4) | | | |
| No | 538 (27) | 1987 (65) |  |
| Yes | 326 (31) | 1058 (35) | 0.03 |
| ^a^ Construction Skills Certification Scheme | | | |

**Table S2 Cox model Results**

| No. of subjects = 3,161 |  |  |  |  |  |  |
| --- | --- | --- | --- | --- | --- | --- |
| Number of observations = 3,161 |  |  |  |  |  |  |
| Log likelihood = -7033.6146 |  |  |  |  |  |  |
| Prob > chi2 = 0.0000 |  |  |  |  |  |  |
| Cox regression |  |  |  |  |  |  |
|  |  |  |  |  |  |  |
| Variables | Haz. Ratio | Std. Err.  (coefficient) | Z | P>\|z\| | 95% CI |  |
|  |  |  |  |  |  |  |
| Female | 1.09 | 0.0896099 | 1.08 | 0.28 | 0.930 | 1.283 |
| Ever worked | 1.54 | 0.1507305 | 4.44 | <0.001 | 1.274 | 1.868 |
| Working at baseline | 0.92 | 0.1010084 | -0.77 | 0.444 | 0.741 | 1.140 |
| Has a long-term job goal | 0.95 | 0.0766363 | -0.63 | 0.527 | 0.811 | 1.113 |
| Restriction in total working hours/days | 0.76 | 0.0626961 | -3.32 | 0.001 | 0.647 | 0.894 |
| Able to drive | 1.14 | 0.1026000 | 1.51 | 0.131 | 0.960 | 1.364 |
| Has a physical or mental health problem | 0.94 | 0.0685142 | -0.91 | 0.361 | 0.810 | 1.079 |
| Using non-prescribed drugs | 0.56 | 0.1308911 | -2.5 | 0.013 | 0.349 | 0.881 |
| Drinks alcohol | 1.09 | 0.0932437 | 1.06 | 0.288 | 0.926 | 1.293 |
| Criminal record | 1.19 | 0.1211973 | 1.75 | 0.079 | 0.979 | 1.457 |
| Stable accommodation | 0.97 | 0.0911566 | -0.29 | 0.774 | 0.810 | 1.169 |
| Caring responsibilities | 1.13 | 0.0931967 | 1.49 | 0.137 | 0.961 | 1.328 |
| Family member/s with drug/alcohol dependency | 1.09 | 0.3067703 | 0.32 | 0.748 | 0.631 | 1.895 |
| Antisocial behaviour issues in family/household | 0.91 | 0.1635495 | -0.53 | 0.599 | 0.639 | 1.294 |
| Domestic abuse family/household | 1.49 | 0.2400740 | 2.49 | 0.013 | 1.088 | 2.045 |
| Customer/family has financial difficulties | 1.07 | 0.0995856 | 0.71 | 0.480 | 0.889 | 1.282 |
| Any other concerns not otherwise disclosed | 0.98 | 0.1044272 | -0.2 | 0.841 | 0.794 | 1.206 |
| Able to read and do everyday maths | 0.88 | 0.1170483 | -0.93 | 0.352 | 0.682 | 1.145 |
| Able to use a computer to apply for work | 1.10 | 0.1198792 | 0.89 | 0.373 | 0.890 | 1.363 |
| Basic industry standard needed | 0.97 | 0.0794815 | -0.31 | 0.754 | 0.830 | 1.143 |
| Confidence Issues | 1.12 | 0.0910633 | 1.36 | 0.174 | 0.952 | 1.310 |
| Difficulties finding/applying for suitable jobs | 0.90 | 0.0751894 | -1.23 | 0.218 | 0.766 | 1.062 |
| Has a CV | 1.40 | 0.1178636 | 4.01 | <0.001 | 1.188 | 1.652 |
| Has clothes for interview | 1.11 | 0.0963784 | 1.26 | 0.209 | 0.940 | 1.320 |
| Would consider self-employment | 0.64 | 0.0799202 | -3.54 | <0.001 | 0.505 | 0.821 |
| Request financial assessment to show financial benefit from working | 0.84 | 0.0648736 | -2.22 | 0.026 | 0.724 | 0.980 |
| Concerns about how to manage money | 0.91 | 0.1417795 | -0.63 | 0.528 | 0.666 | 1.231 |
| Able to attend appointments and meetings | 1.21 | 0.3118392 | 0.74 | 0.462 | 0.729 | 2.004 |
| Able to mix and work well with colleagues | 0.77 | 0.1244091 | -1.63 | 0.104 | 0.559 | 1.055 |
| Travel to work <1 hour | 1.04 | 0.1139969 | 0.31 | 0.753 | 0.834 | 1.284 |
| Travel to work >1 hour | 1 | Omitted |  |  |  |  |
| Has caring responsibilities were alternative arrangements needed | 0.64 | 0.0604697 | -4.73 | <0.001 | 0.531 | 0.769 |
| Support needed whilst at work | 1.13 | 0.1120242 | 1.22 | 0.223 | 0.929 | 1.370 |
| Is financial help needed to start work | 1.30 | 0.1053065 | 3.22 | 0.001 | 1.107 | 1.522 |
| Homeless | 0.71 | 0.1238972 | -1.94 | 0.053 | 0.509 | 1.004 |
| Age group |  |  |  |  |  |  |
| 16 to 25 | 1.50 | 0.2338114 | 2.6 | 0.009 | 1.104 | 2.035 |
| 26 to 35 | 1.38 | 0.1995640 | 2.2 | 0.027 | 1.036 | 1.828 |
| 36 to 45 | 1.42 | 0.2034404 | 2.48 | 0.013 | 1.076 | 1.884 |
| 46 to 55 | 1.08 | 0.1639166 | 0.48 | 0.635 | 0.797 | 1.449 |
| 56 to 69 | 1 | (omitted) |  |  |  |  |
| Ethnicity |  |  |  |  |  |  |
| White | 0.84 | 0.0901876 | -1.61 | 0.107 | 0.681 | 1.037 |
| Black | 0.87 | 0.0909734 | -1.3 | 0.195 | 0.712 | 1.071 |
| Asian | 0.82 | 0.0965263 | -1.72 | 0.085 | 0.646 | 1.028 |
| unknown/other | 1 | (omitted) |  |  |  |  |
| Education |  |  |  |  |  |  |
| Primary | 1.65 | 0.5996713 | 1.39 | 0.165 | 0.813 | 3.366 |
| Lower Secondary | 1.34 | 0.4180035 | 0.95 | 0.341 | 0.731 | 2.473 |
| Upper Secondary | 1.48 | 0.4581534 | 1.27 | 0.205 | 0.806 | 2.715 |
| Post Secondary | 1.26 | 0.3972591 | 0.75 | 0.455 | 0.683 | 2.340 |
| Tertiary | 1.70 | 0.5418230 | 1.68 | 0.094 | 0.913 | 3.177 |
| Living situation |  |  |  |  |  |  |
| Lives alone | 0.83 | 0.1061330 | -1.42 | 0.155 | 0.650 | 1.070 |
| Single parent | 1.02 | 0.1893562 | 0.08 | 0.934 | 0.704 | 1.461 |
| With someone | 0.99 | 0.1048555 | -0.09 | 0.932 | 0.805 | 1.219 |
| Children/dependents | 1.22 | 0.1493574 | 1.6 | 0.110 | 0.956 | 1.547 |
| other/unknown | 1 | (omitted) |  |  |  |  |
| Able to travel | 1.10 | 0.0857214 | 1.21 | 0.226 | 0.943 | 1.280 |
| Not able to travel | 1 | Omitted |  |  |  |  |
| Household issues and barriers |  |  |  |  |  |  |
| Additional barriers | 1.63 | 0.1877146 | 4.28 | <0.001 | 1.305 | 2.047 |
| Long term unemployed | 1.74 | 0.2203426 | 4.4 | <0.001 | 1.361 | 2.233 |
| Involved in crime | 0.88 | 0.1200213 | -0.91 | 0.364 | 0.677 | 1.153 |
| Unknown or other barrier | 1 | (omitted) |  |  |  |  |
| London Borough |  |  |  |  |  |  |
| East | 1.25 | 0.2158460 | 1.27 | 0.203 | 0.887 | 1.750 |
| North | 1.18 | 0.2268308 | 0.84 | 0.399 | 0.806 | 1.716 |
| Outer | 1 | (omitted) |  |  |  |  |

**Table S3: Royston-Parmar Results**

| Log likelihood = -3215.7022 Number of observations = 3,161 | | | | | |  |
| --- | --- | --- | --- | --- | --- | --- |
|  |  |  |  |  |  |  |
|  |  |  |  |  |  |  |
| Variables | HR | Std. Err. | z | P>\|z\| | 95% Conf. Interval | |
|  |  |  |  |  |  |  |
|  |  |  |  |  |  |  |
| Female | 1.09191 | 0.0895519 | 1.07 | 0.284 | 0.9297722 1.282323 |  |
| Ever worked | 1.544476 | 0.1508926 | 4.45 | <0.001 | 1.275323 1.870433 |  |
| Working at baseline | 0.9194014 | 0.101027 | -0.76 | 0.444 | 0.7412627 1.14035 |  |
| Has a long-term job goal | 0.9496909 | 0.0765827 | -0.64 | 0.522 | 0.8108521 1.112302 |  |
| Restriction in total working hours/days | 0.7596332 | 0.0626151 | -3.34 | 0.001 | 0.6463102 .8928261 |  |
| Able to drive | 1.14505 | 0.1026238 | 1.51 | 0.131 | 0.9605861 1.364936 |  |
| Has a physical or mental health problem | 0.9354522 | 0.0685511 | -0.91 | 0.363 | 0.8102974 1.079938 |  |
| Using non-prescribed drugs | 0.5555336 | 0.1309128 | -2.49 | 0.013 | 0.3500441 .8816535 |  |
| Drinks alcohol | 1.093417 | 0.093145 | 1.05 | 0.294 | 0.9252824 1.292103 |  |
| Criminal record | 1.190785 | 0.1207744 | 1.72 | 0.085 | 0.9761145 1.452666 |  |
| Stable accommodation | 0.9728015 | 0.0910894 | -0.29 | 0.768 | 0.809694 1.168766 |  |
| Caring responsibilities | 1.130049 | 0.0931759 | 1.48 | 0.138 | 0.9614198 1.328255 |  |
| Family member/s with drug/alcohol dependency | 1.089607 | 0.3054385 | 0.31 | 0.759 | 0.6290153 1.887464 |  |
| Antisocial behaviour issues in family/household | 0.9097674 | 0.1635456 | -0.53 | 0.599 | 0.6396066 1.29404 |  |
| Domestic abuse family/household | 1.496785 | 0.2409112 | 2.51 | 0.012 | 1.091833 2.051929 |  |
| Customer/family has financial difficulties | 1.069613 | 0.0997218 | 0.72 | 0.47 | 0.8909798 1.284061 |  |
| Any other concerns not otherwise disclosed | 0.9779344 | 0.104338 | -0.21 | 0.834 | 0.7934016 1.205387 |  |
| Able to read and do everyday maths | 0.885342 | 0.1171965 | -0.92 | 0.358 | 0.6830207 1.147594 |  |
| Able to use a computer to apply for work | 1.100579 | 0.1197577 | 0.88 | 0.378 | 0.8891998 1.362208 |  |
| Basic industry standard needed | 0.9758552 | 0.0795764 | -0.3 | 0.764 | 0.8317138 1.144977 |  |
| Confidence Issues | 1.118777 | 0.0911947 | 1.38 | 0.169 | 0.9535859 1.312585 |  |
| Difficulties finding/applying for suitable jobs | 0.9009833 | 0.0750549 | -1.25 | 0.211 | 0.7652597 1.060778 |  |
| Has a CV | 1.399777 | 0.1177102 | 4.00 | <0.001 | 1.187078 1.650586 |  |
| Has clothes for interview | 1.114054 | 0.0963197 | 1.25 | 0.212 | 0.9403998 1.319776 |  |
| Would consider self-employment | 0.6442053 | 0.0799141 | -3.54 | <0.001 | 0.5051637 0.8215168 |  |
| Request financial assessment to show financial benefit from working | 0.8418492 | 0.0647993 | -2.24 | 0.025 | 0.723961 .9789341 |  |
| Concerns about how to manage money | 0.9072573 | 0.142007 | -0.62 | 0.534 | 0.6675711 1.233001 |  |
| Able to attend appointments and meetings | 1.21149 | 0.3124801 | 0.74 | 0.457 | 0.73075 2.008494 |  |
| Able to mix and work well with colleagues | 0.7698604 | 0.1246263 | -1.62 | 0.106 | 0.5605548 1.057319 |  |
| Travel to work>1 hour | 1.035779 | 0.1140566 | 0.32 | 0.75 | 0.8347102 1.285283 |  |
| Travel to work < 1hour | 1 | Omitted |  |  |  |  |
| Has caring responsibilities were alternative arrangements needed | 0.6400262 | 0.060538 | -4.72 | <0.001 | 0.5317229 0.7703891 |  |
| Support needed whilst at work | 1.128183 | 0.1119739 | 1.22 | 0.224 | 0.9287452 1.370448 |  |
| Is financial help needed to start work | 1.298179 | 0.1052797 | 3.22 | 0.001 | 1.107398 1.521827 |  |
| Homeless | 0.7148738 | 0.12387 | -1.94 | 0.053 | 0.5090226 1.003972 |  |
| Age group |  |  |  |  |  |  |
| 16 to 25 | 1.498472 | 0.233718 | 2.59 | 0.010 | 1.103789 2.034283 |  |
| 26 to 35 | 1.37718 | 0.1996655 | 2.21 | 0.027 | 1.036531 1.829782 |  |
| 36 to 45 | 1.42471 | 0.2035251 | 2.48 | 0.013 | 1.076785 1.885054 |  |
| 46 to 55 | 1.075357 | 0.1639413 | 0.48 | 0.634 | 0.7975989 1.449842 |  |
| 56 to 69 | 1 | (omitted) |  |  |  |  |
| Ethnicity |  |  |  |  |  |  |
| White | 0.8423531 | 0.0902986 | -1.60 | 0.110 | 0.682727 1.039301 |  |
| Black | 0.8747422 | 0.0910708 | -1.29 | 0.199 | 0.7132802 1.072754 |  |
| Asian | 0.8150948 | 0.0964992 | -1.73 | 0.084 | 0.6463001 1.027974 |  |
| unknown/other | 1 | (omitted) |  |  |  |  |
| Education |  |  |  |  |  |  |
| Primary | 1.653133 | 0.5991365 | 1.39 | 0.165 | 0.8124769 3.3636 |  |
| Lower Secondary | 1.34312 | 0.4174156 | 0.95 | 0.343 | 0.7304289 2.469743 |  |
| Upper Secondary | 1.477337 | 0.4572019 | 1.26 | 0.207 | 0.8054753 2.709611 |  |
| Post Secondary | 1.26291 | 0.3966561 | 0.74 | 0.457 | 0.6823795 2.337322 |  |
| Tertiary | 1.700204 | 0.5405028 | 1.67 | 0.095 | 0.9118013 3.170312 |  |
| Living situation |  |  |  |  |  |  |
| Lives alone | 0.835946 | 0.1063154 | -1.41 | 0.159 | 0.6515124 1.07259 |  |
| Single parent | 1.017816 | 0.1897886 | 0.09 | 0.925 | 0.7062344 1.466864 |  |
| With someone | 0.9928224 | 0.1050583 | -0.07 | 0.946 | 0.806862 1.221642 |  |
| Children/dependents | 1.217505 | 0.1494211 | 1.6 | 0.109 | 0.9572052 1.54859 |  |
| other/unknown | 1 | (omitted) |  |  |  |  |
| Able to travel | 1.099838 | 0.0857776 | 1.22 | 0.222 | 0.9439362 1.281489 |  |
| Not able to travel | 1 | omitted |  |  |  |  |
| Household issues and barriers |  |  |  |  |  |  |
| long term unemployed | 1.62308 | 0.1864176 | 4.22 | <0.001 | 1.295913 2.032844 |  |
| Involved in crime | 1.727097 | 0.2183085 | 4.32 | <0.001 | 1.348104 2.212638 |  |
| Unknown or other barrier | 0.8856099 | 0.1202437 | -0.89 | 0.371 | 0.6786886 1.155618 |  |
| Household issues and barriers | 1 | (omitted) |  |  |  |  |
| London Borough |  |  |  |  |  |  |
| East | 1.243714 | 0.215392 | 1.26 | 0.208 | 0.8857386 1.746367 |  |
| North | 1.176138 | 0.2267391 | 0.84 | 0.400 | 0.8060486 1.71615 |  |
| Outer | 1 | (omitted) |  |  |  |  |

**Table S4 Ranking of variables using different analytical approaches**

| Rank | Cox and Royston Parmar  [Importance based on Regression Coefficient] | Conditional Survival Forest [score feature importance] (percent feature importance) | Cox Elastic Net* |
| --- | --- | --- | --- |
| 1 | Ever Worked[1.48] | Working hours restricted  [0.104] (5.265) | Domestic abuse family/household |
| 2 | Financial help needed to start work [1.36] | Caring responsibilities that need alternative arrangements  [0.096] (4.832) | Self employment considered |
| 3 | Domestic abuse family/household [1.51] | Has CV  [0.089] (4.477) | Primary education |
| 4 | Has CV [1.42] | Unknown or other barrier  [0.077] (3.854) | Caring responsibilities that need alternative arrangements |
| 5 | Age 16-25 [1.53] | Ever worked  [0.074] (3.727) | Using non-prescribed drugs |
| 6 | Age 26-35 [1.39] | Able to travel  [0.043] (2.154) |  |
| 7 | Age 36-45[1.46] | Consider self-employment  [0.033] (1.671) |  |
| 8 | Primary Education[0.48] | Tertiary Education  [0.032] (1.644) |  |
| 9 | Tertiary education[0.76] | Not able to travel  [0.032] (1.635) |  |
| 10 | Unknown or other barrier[0.60] | Drinks Alcohol  [0.0311] (1.562) |  |
| 11 | Using non-prescribed drugs [0.55] |  |  |
| 12 | Consider self-employment [0.65] |  |  |
| 13 | Caring responsibilities [0.61] |  |  |
| 14 | Working hours restricted[0.76] |  |  |
| *Variable importance see figure S1 | | | |

Figure S1 Output from Cox Elastic net


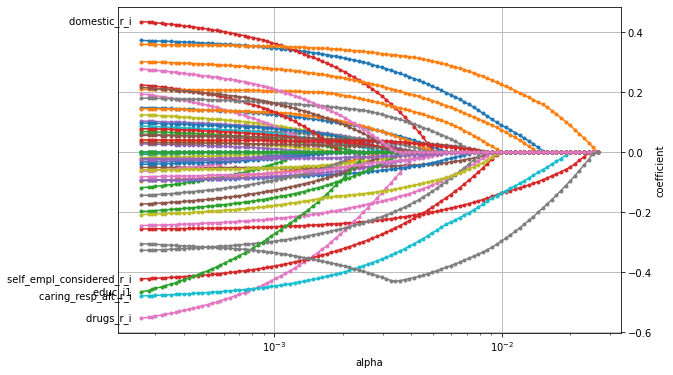


Variables for Figure 1 from paper

var1, Caring responsibilities; var2, Unknown or other barrier; var3, Willing to consider self-employment; var4, Restrictions to working hours; var5, Using non-prescribed drugs; var6, Age 56-69; var7, age 46-55; var8, Lives alone; var9, Homeless; var10, Concerns about how ot manage money; var11, Difficulties finding/applying for suitable jobs; var12, Request financial assessment to show financial benefit from working ; var13, Working at baseline; var14, Basic industry standard needed; var15, Physical or mental health issues; var16, Antisocial behaviour issues in family/household; var17, Asian; var18, Primary Education; var19, Outer London; var20, Able to read and do everyday maths; var21, Single parent; var22, Has a long-term job goal; var23, Family member/s with drug/alcohol dependency ; var24, Unable to travel >1hour; var25, Secondary education; var26, Can use computer; var27, Post Secondary Education ; var28, Age 36-45; var29, East London; var30, Black ; var31, Financial difficulties; var32, Suitable clothes; var33, Able to travel >1 hour; var34, Age 16 to 25; var35, Drinks alcohol; var36, Concerns about travel to work; var37, Able to make appointments; var38, Does not need support at work; var39, Will mix well at work; var40, No confidence issues; var41, North London; var42, Can Drive; var43, Not criminal record; var44, Ethnicity - other; var45, Caring responsibilities; var46, Domestic abuse; var47, Lives with Children; var48, No additional barriers; var49, Not long term unemployed ; var50, Needs practical financial help; var51, Ever worked; var52, Has a CV
